# A bivalent live-attenuated vaccine candidate elicits protective immunity against human adenovirus types 4 and 7

**DOI:** 10.1080/22221751.2021.1981157

**Published:** 2021-09-27

**Authors:** Jingao Guo, Youbin Zhang, Yan Zhang, Chao Zhang, Caihong Zhu, Man Xing, Xiang Wang, Dongming Zhou

**Affiliations:** a University of Chinese Academy of Sciences, Beijing, People’s Republic of China; b Chinese Academy of Sciences, Institut Pasteur of Shanghai, Shanghai, People’s Republic of China; c Department of Emergency Surgery, First Hospital of Soochow University, Suzhou, People’s Republic of China; d School of Medicine & Holistic Integrative Medicine, Nanjing University of Chinese Medicine, Nanjing, People’s Republic of China; e Shanghai Public Health Clinical Center, Fudan University, Shanghai, People’s Republic of China; f Department of Pathogen Biology, School of Basic Medical Sciences, Tianjin Medical University, Tianjin, People’s Republic of China

**Keywords:** HAdV4, HAdV7, live-attenuated viruses, vaccine, immune response

## Abstract

Human adenovirus types 4 (HAdV4) and 7 (HAdV7) often lead to severe respiratory diseases and occur epidemically in children, adults, immune deficiency patients, and other groups, leading to mild or severe symptoms and even death. However, no licensed adenovirus vaccine has been approved in the market for general use. E3 genes of adenovirus are generally considered nonessential for virulence and replication; however, a few studies have demonstrated that the products of these genes are also functional. In this study, most of the E3 genes were deleted, and two E3-deleted recombinant adenoviruses (ΔE3-rAdVs) were constructed as components of the vaccine. After E3 deletion, the replication efficiencies and cytopathogenicity of ΔE3-rAdVs were reduced, indicating that ΔE3-rAdVs were attenuated after E3 genes deletion. Furthermore, single immunization with live-attenuated bivalent vaccine candidate protects mice against challenge with wild-type human adenovirus types 4 and 7, respectively. Vaccinated mice demonstrated remarkably decreased viral loads in the lungs and less lung pathology compared to the control animals. Taken together, our study confirms the possibility of the two live-attenuated viruses as a vaccine for clinic use and illustrates a novel strategy for the construction of an adenovirus vaccine.

## Introduction

Human adenovirus (HAdV) is classified in the genus *Mastadenovirus,* which contains seven subgroups from A to G. At present, at least 90 types of human adenoviruses have been isolated. HAdV shows a broad range of tissue tropisms, which often depend on the type of HAdV [1]. Common clinical diseases caused by HAdV infection include acute respiratory disease (ARD), conjunctivitis, gastroenteritis, hepatitis, myocarditis, pneumonia, and central nervous system diseases [2–4]. Most of these diseases are self-limiting or mild. However, in some cases, patients infected by HAdV can become severe and require intensive care, even worse, disability and death [5,6]. HAdV pandemics occur globally and mostly occur in winter or early spring; however, epidemics can occur throughout the year with no clear seasonality [2]. At least 5–10% of pediatric and 1–7% of adult respiratory tract infections (RTIs) can be attributed to HAdV infection worldwide. Patients who received hematopoietic stem cell transplantation (HSCT) and solid organ transplant (SOT) have a higher risk of death [7,8].

Among these, human adenovirus type 4 (HAdV4) and 7 (HAdV7), which can be categorized as type E and B, respectively [9,10], have occurred epidemically and caused outbreaks in China and several other countries and continents, sometimes affecting patient survival [5,11–14]. A systematic review of scientific reports of HAdV4 infections between January 1960 and November 2020 indicated that there had been a continuous increase in civilian HAdV4 infections over the past decade [15]. Other studies have highlighted that HAdV7 infection caused more severe pneumonia than HAdV3 and other HAdV types [16,17]. However, recombination and variation of the viral genome enable HAdV4 and 7 more potential outbreaks in immune-naïve civilian populations [14,17]. Consequently, effective preventive approaches and vaccine interventions for adenovirus infection are urgently needed.

Before the 1970s, HAdV4 and HAdV7 were the major causes of ARD in US military trainees [18] and affected 80% of new recruits during recruitment training; 40% showed severe symptoms, and 20% required hospitalization. These diseases cause a great burden, including a large cost for medical care and severe disruption of the troop training programme [18–20]. Consequently, in 1971, a live oral vaccine against HAdV4 and HAdV7 was introduced for new recruits [21]. Vaccine administration in the military dramatically reduced the incidence of adenovirus-related respiratory febrile illness. The unattenuated adenovirus vaccine production was discontinued in 1996 [22], despite vaccination being an efficient strategy against adenovirus circulation. By 1999, vaccine stocks were depleted; as a result, the live adenovirus vaccine was suspended [23]. From 1999 to 2010, outbreaks of adenovirus-associated ARD returned to the pre-vaccine level, and eight deaths were reported related to adenovirus infection in the US military during this period [24]. The reemerged adenovirus infection forced the US military to reuse the adenovirus vaccine since 2011 [23]. After the first two years of universal vaccination in the military, the adenovirus-associated disease burden decreased approximately 100-fold among recruits to an incidence of effectively zero [25,26]. Vaccination has become a cost-effective strategy for the prevention of adenovirus infection.

Although the live adenovirus vaccine has shown its effectiveness, new adenovirus vaccines still need to be developed due to positive viral shedding and an unexpected higher rate of adverse effects [27,28]. Comprehensive attention has been paid to live attenuated vaccines (LAVs) since they can mimic natural viral infections to elicit immune responses. Meanwhile, a single administration of LAVs can stimulate a robust, fast, and durable immune response efficiently, although sometimes with the trade-off of reduced safety [29]. However, other vaccines, such as subunit vaccines, VLPs vaccines, and DNA vaccines, have drawbacks, including low immunogenicity, adjuvant needs, and periodic boosters [30–32].

This study constructed two E3-deleted recombinant adenoviruses (ΔE3-rAdVs) ΔE3-HAdV4 and ΔE3-HAdV7 and characterized the effects of E3 genes deletion on the replication and cytopathogenicity of rAdVs compared with wild-type adenovirus (Wt-AdVs). We also demonstrated the potential of ΔE3-rAdVs as a live-attenuated adenovirus vaccine and provided a new strategy for adenovirus vaccine development.

## Materials and methods

### Cells and viruses

HEK293 (human embryonic kidney cells) and A549 (human lung carcinoma cells) cell lines were maintained in complete Dulbecco’s modified Eagle’s medium supplemented with 10% fetal bovine serum (FBS, Gibco) and 2% penicillin and streptomycin (New Cell & Molecular Biotech) and incubated at 37°C and 5% CO_2_. Wild-type human adenovirus 4 (Wt-HAdV4, GenBank: AY594253) and wild-type human adenovirus 7 (Wt-HAdV7, GenBank: AY594255) were purchased from ATCC.

### Construction and production of recombinant E3-deleted adenovirus

E3 regions deleted- recombinant adenovirus (ΔE3-rAdVs) were constructed by direct cloning and isothermal assembly. Wild-type and recombinant adenoviruses were rescued, propagated, and purified by CsCl gradient ultracentrifugation, as previously described [33]. Viral particles (VPs) were calculated using spectrophotometry. Plaque forming units (PFUs) were determined by plaque assay as those previously described [33,34]. Isothermal assembly kits were purchased from New Cell & Molecular Biotech, and restriction endonucleases were purchased from New England Biolabs.

### Genetic stability test

For genetic stability test, two recombinant adenoviruses (ΔE3-rAdVs) were passaged continuously and the 12th generation viruses were purified, respectively. 100 µl (∼5 × 10^11^vp) of each purified virus was used for preparation of genomic DNA according to the manufacturer’s instructions of DNeasy Tissue Kit (QIAGEN). Then, 1.0 µg of ΔE3-HAdV4 genomic DNA was digested with 3 restriction enzymes individually including BamHI, XhoI, and MfeI, and 1.0 µg of ΔE3-HAdV7 genomic DNA was digested by BglI, XhoI, and XmaI*,* respectively. All the enzymes digestion was performed at 37°C for 3 h. 1% agarose gel was prepared for electrophoresis.

### Western blot

**Figure 1.**
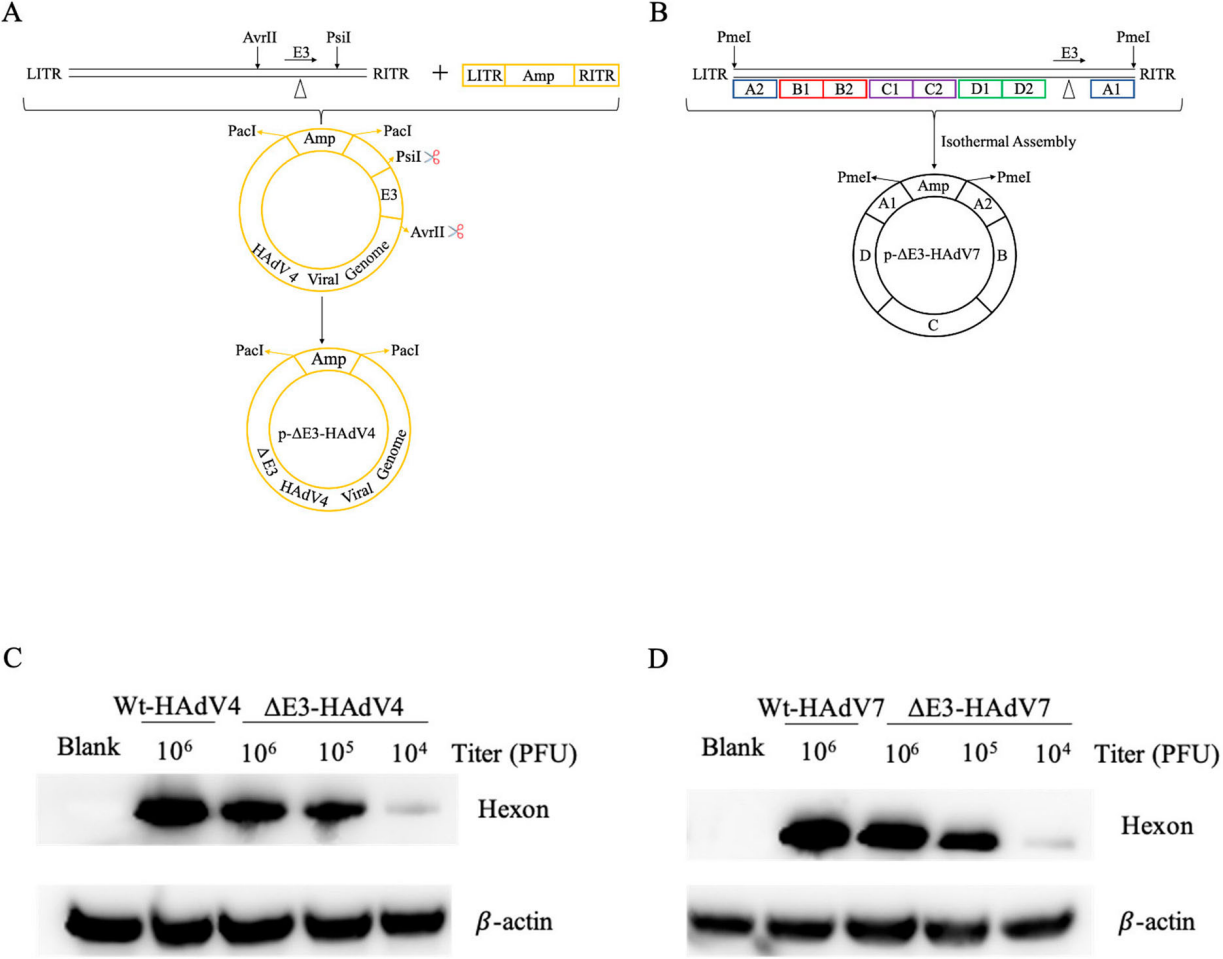
Construction of ΔE3-HAdV4 and ΔE3-HAdV7. Diagrams of construction of ΔE3-HAdV4 (A) and ΔE3-HAdV7 (B) from wild type adenovirus. LITR, left inverted terminal repeat; RITR, right inverted terminal repeat. (C and D) are western blot assay results. Total protein extracts of HEK293 cells infected with Wt-HAdV4, Wt-HAdV7, ΔE3-HAdV4 and ΔE3-HAdV7 in gradient concentration were analyzed by western blot under non-reducing condition using anti-HAdV4 hexon antibody (C) and anti-HAdV7 hexon antibody (D), respectively.

HEK293 cells were seeded into 6-well plates in advance and then infected with ΔE3-AdVs at concentrations of 10^6^, 10^5^, and 10^4^ PFU/well. 10^6^ PFU of Wt-AdVs and PBS were used as positive and negative controls, respectively. At 24h after infection, cells were collected and lysed in 100 ul RIPA buffer containing protease inhibitors. Cell lysates were analyzed by western blotting with anti-HAdV7 hexon monoclonal antibody customized by Abcam (Figure 1D) and anti-HAdV4 hexon (Figure 1C) monoclonal antibody (provided by Zhong HUANG’s lab, Institut Pasteur of Shanghai, China), respectively. For cross-reaction test between ΔE3-HAdV4 and ΔE3-HAdV7, the sera containing polyclonal antibodies from mice immunized with ΔE3-HAdV4 (Figure 4A) or ΔE3-HAdV7 (Figure 4B) were used in western blotting detection. Anti-β-actin was detected as a loading control.

### ELISA

96-well ELISA plates were coated with 10^10^ vp/100 µl/well of Wt-AdVs at 4°C overnight. The viruses were inactivated at 56°C for 30 min before the coating. After blocking with 200 μL of 10% skim milk at 37°C for 2 h, serially diluted serum samples were added and incubated at 37°C for 2 h. 1:5000 dilutions of horseradish peroxidase (HRP)-conjugated anti-mouse IgG secondary antibody (Sigma–Aldrich) was added and incubated at 37°C for 1 h. Subsequently, 50 μL of TMB substrate (New Cell & Molecular Biotech) was added to each well. Thereafter, 50 μL of 2 M H_2_SO_4_ solution was added to halt the reaction. Absorbance at 450 nm was measured using a Varioskan Flash multimode ELISA plate reader (Thermo Scientific). The antibody endpoint titre was determined as the highest serum dilution whose absorbance was 0.1 optical density (OD) unit above the control of PBS-treated samples.

### Neutralization assay

50 μL 2-fold serial dilutions of serum samples (starting at a 1:200 dilution) were prepared and mixed with 100 TCID50 of Wt-AdVs in the same volume for each well of 96-well cell culture plates. The mixture was incubated at 37°C for 1 h. Subsequently, 2 × 10^4^ HEK293 cells were added to each well and incubated at 37°C for 7 days. Neutralization titres were defined as the dilution at which a 50% reduction in CPE was observed relative to the virus alone and PBS alone.

### In vitro replicative capacity

The growth characteristics were compared to those of wild-type adenovirus in the A549 cell line. Cells were infected with 0.01 MOI of Wt-HAdV4 or ΔE3-HAdV4 and 0.1 MOI of Wt-HAdV7 or ΔE3-HAdV7 and then harvested at 12, 24, 36, 48, 72, and 96 h post-infection, respectively. Viral genomic DNA copies at each time point were obtained using the DNeasy Tissue Kit (QIAGEN) and determined by quantitative real-time PCR (qRT-PCR) (SYBR™ Green PCR Master Mix, Thermo Scientific) according to the manufacturer’s instructions. The plasmids containing the whole genome of Wt-AdVs were serially 10-fold diluted (10^1^–10^9^ copies) as a standard for the determination of genomic DNA copies.

### CCK8 assay

A549 cells were seeded into 96-well plates at a density of 10^5^ cells per well 24 h before detecting the cytotoxic effect of the ΔE3-AdVs. Various MOIs of viruses (0.1, 0.3, and 1 MOI for Wt-HAdV4 or ΔE3-HAdV4 and 0.3, 1, 3 MOI for Wt-HAdV7 or ΔE3-HAdV7) were added to each well. Cell viability (%) was detected using the CCK8 kit (New Cell & Molecular Biotech) every 24 h until 96 h after infection.

### Plaque assay

HEK293 cells were seeded into 6-well plates at a density of 10^6^ cells/well 24 h before the plaque assay. Thereafter, 0.01 MOI of Wt-HAdV4 or ΔE3-HAdV4 and 0.1 MOI of Wt-HAdV7 or ΔE3-HAdV7 were added. Cell lysates and supernatants collected at different time points were added to individual wells containing confluent cells. The plates were incubated at 37°C with 5% CO_2_ for 1 h before adding a layer of 0.4% low-melt agarose diluted in 2% FBS DMEM. After 7 days of incubation, the cells were fixed with 4% paraformaldehyde solution for 2 h and stained with 1% crystal violet for 1 h. Fix staining was performed at room temperature. Plaque morphology and number were recorded after washing with tap water.

### Animal study

Female BALB/c mice (6–8 weeks of age) were obtained from the Shanghai Laboratory Animal Center and divided randomly into different groups (5 or 6 mice/group). Mice were intramuscularly (i.m.) immunized with a total amount of 10^6^ PFU/100 μl/mouse of ΔE3-AdVs or Wt-AdVs. Control mice were injected with the same volume of phosphate-buffered saline (PBS). Blood samples were collected and analyzed by ELISA and neutralization tests. Four weeks after inoculation, mice were challenged intranasally with 10^6^ PFU/mouse of Wt-HAdV4 or 2 × 10^6^ PFU/mouse of Wt-HAdV7. Five days or three days after Wt-HAdV4 or Wt-HAdV7 attack, mice were sacrificed, and the lung tissues were collected to examine the viral genome copies by qRT-PCR. Hexon gene (Forward primer: ACCAGCTCTTGCTTGACTCT; Reverse primer: GGCAATTCATCCTCCACACC) of Wt-HAdV4 and E1B gene (Forward primer: CGTTTCACATGCACGCAAGA; Reverse primer: GGCATAAACATTCCCCTGCG) of Wt-HAdV7 were included as target genes. The viral loads of each sample were converted using the Ct value and the standard curve as described above.

For tissue distribution assay, 6–8 weeks old female BALB/c mice were divided into 4 or 5 per group. Mice were infected intranasally with 10^6^ PFU/mouse of Wt-/ΔE3-HAdV4 or 2 × 10^6^ PFU/mouse of Wt-/ΔE3-HAdV7. Tissue samples including liver, spleen, kidney, ovary, lung, heart, trachea, brain and blood were harvested at 3 and 7 days after Wt-/ΔE3-HAdV7 challenge, and 5 and 10 days after Wt-/ΔE3-HAdV4 infection, respectively. Viral genome copies were determined by qRT-PCR as described above.

All animal experimental protocols were reviewed and approved by the Institutional Animal Care and Use Committee of the Institute Pasteur of Shanghai.

### Histological observation

Lung samples were fixed in 4% paraformaldehyde solution for 24 h at 4°C and subjected to hematoxylin and eosin staining for histological observation. Pathological scores in the lungs were determined according to the following criteria: 1. No pathological changes were observed; 2. slightly perivascular infiltrates were observed; 3. perivascular and interstitial infiltrates were observed in <20% of the lobe sections; 4. Perivascular and interstitial infiltrates were observed in 20–50% of the lobe sections; 5. Perivascular and interstitial infiltrates were observed in >50% of the lobe sections.

### Statistical analysis

GraphPad Prism (GraphPad software 7.0) was used for statistical analysis and to plot data. Statistical significance was set at *P* < 0.05.

## Results

### Construction of ΔE3-HAdV4 and ΔE3-HAdV7

Previous studies have shown that E3 genes of adenovirus are related to interaction with host immunity; however, they are not necessary for virion assembly, replication, and infectious capacity [35,36]. As a result, the E3 genes are deleted to increase the foreign gene insert capacity when used as a vaccine vector [37]. Therefore, we assumed that deletion of the E3 genes from Wt-HAdV4 and 7 does not affect the formation and propagation of viral particles. By using direct cloning and isothermal assembly methods, we successfully constructed two E3-deleted recombinant adenoviral vectors, ΔE3-HAdV4 and ΔE3-HAdV7 plasmids (Figure 1A,B). For type 4, 91.7% sequences from 27,323 to 31,383 of the E3 region were deleted. Approximately 88.1% sequences from 27,538 to 31,122 of the E3 part were removed from the type 7 genome.

Among the three major structural proteins (hexon, fibre, and penton), hexon is the predominant target of serotype-specific neutralizing antibodies (NAbs) [38,39]. The western blot was performed with anti-HAdV4 or HAdV7 hexon monoclonal antibodies, indicating that hexon proteins of ΔE3-HAdV4 and ΔE3-HAdV7 are expressed normally (Figure 1C,D). In addition, two E3-deleted recombinant viruses remain stable without any detectable genetic instability after passage 12 generations (Figure S1).

### Characterizations of ΔE3-HAdV4 and ΔE3-HAdV7

**Figure 2.**
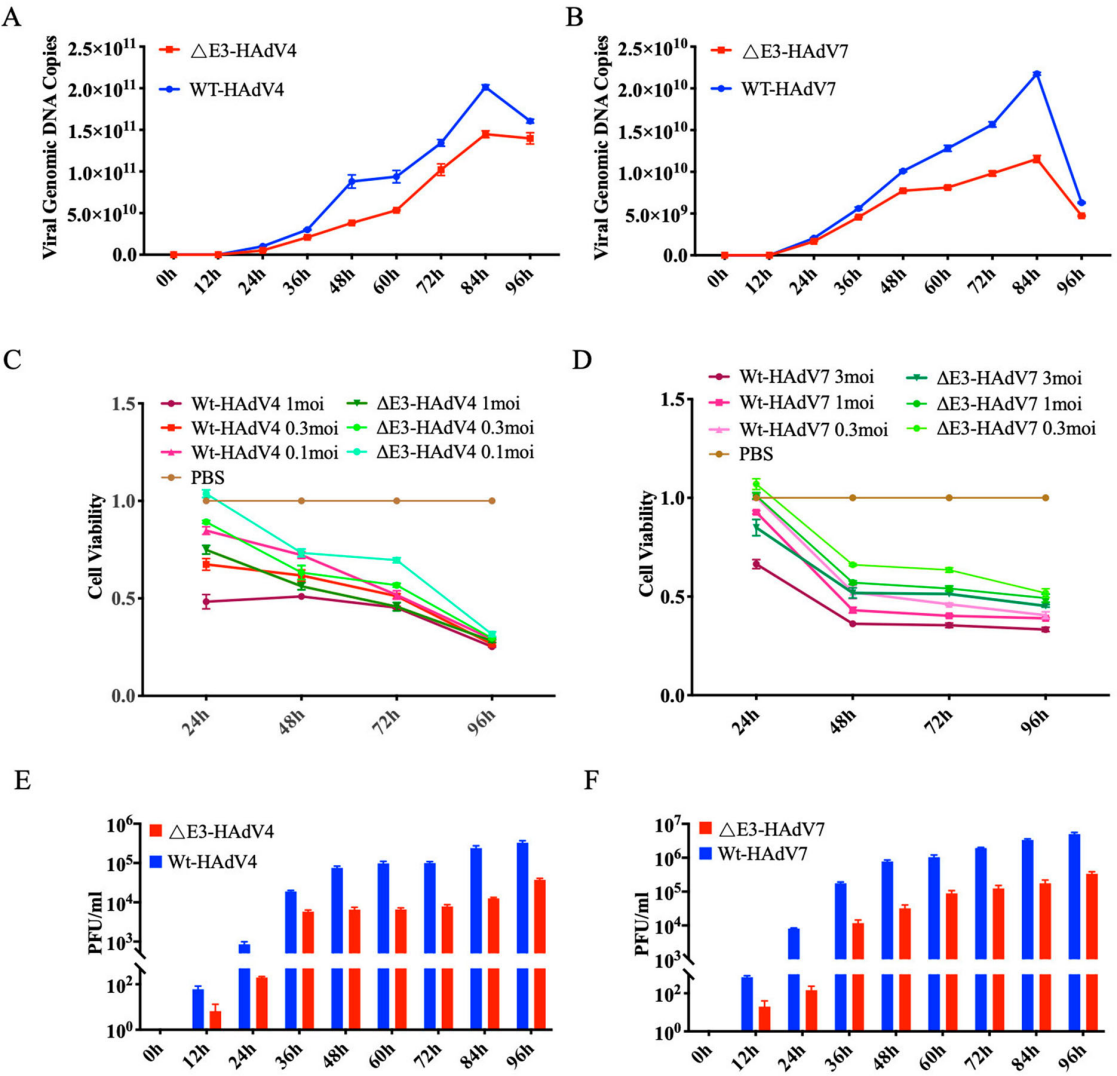
Characterization of ΔE3-HAdV4 and ΔE3-HAdV7. (A) Comparation of replication capacity between Wt-HAdV4 and ΔE3-HAdV4. Wt-HAdV4 vs. ΔE3-HAdV4, ***, *P* = 0.0002. (B) Comparation of replication capacity between Wt-HAdV7 and ΔE3-HAdV7. Wt-HAdV7 vs. ΔE3-HAdV7, ****, *P* < 0.0001. (C) Comparation of cell viability after Wt-HAdV4 and ΔE3-HAdV4 infection. Wt-HAdV4 vs. ΔE3-HAdV4 at 1moi, ***, *P* = 0.0001; at 0.3 moi, ****, *P* < 0.0001; at 0.1 moi, ***, *P* = 0.0002. (D) Comparation of cell viability after Wt-HAdV7 and ΔE3-HAdV7 infection. Wt-HAdV7 vs. ΔE3-HAdV7 at 1 moi, ****, *P *< 0.0001; at 0.3 moi, ****, *P* < 0.0001; at 0.1 moi, ****, *P* < 0.0001. (E) Comparation of PFU between Wt-HAdV4 and ΔE3-HAdV4. Wt-HAdV4 vs. ΔE3-HAdV4, **, *P* = 0.0014. (F) Comparation of PFU between Wt-HAdV7 and ΔE3-HAdV7. Wt-HAdV7 vs. ΔE3-HAdV7, ****, *P* < 0. 0001. Two-way ANOVA was applied to compare the differences between groups. All data are presented as the means ± SEM. Each experiment was repeated three times. ns, no significance; *, *P* < 0.05; **, *P* < 0.01; ***, *P* < 0.001; ****, *P* < 0.0001.

We wondered if E3 genes deletion would affect the growth characteristics and cytotoxicity of rAdVs. Thus, we tested the growth capacity of E3-deleted rAdVs and Wt-AdVs. Compared to Wt-AdVs, both ΔE3-rAdVs showed slower replication kinetics and lower peak titres at 84 h (Figure 2A,B). The CCK-8 assay was performed for cell viability detection to determine the effects of E3 deletions on cytopathogenicity. At 24 h after infection, cells infected with 1 MOI Wt-HAdV4 showed the most severe damage and induced more than 50% of the cells died, while cells infected with 1 MOI of ΔE3-HAdV4 showed approximately 75% of the cells alive. For 0.01 MOI ΔE3-HAdV4, cell viability was ∼100% at 24 h post infection. At 96 h post infection, the vitality of cells infected with ΔE3-HAdV4 and Wt-HAdV4 was approximately 30% (Figure 2C). Overall, the cell viability of the ΔE3-HAdV4 infection groups was higher than that of Wt-HAdV4, regardless of the infection dose. ΔE3-HAdV7 showed modest cytopathogenicity compared to Wt-HAdV7 in all MOI infections. At 96 h, even the highest dose infection group (3 MOI) showed more than 50% cell survival, and all groups showed the same time and dose-dependent tendency as Wt/ΔE3-HAdV4 (Figure 2D).

To further verify the variation in cytopathogenicity between Wt- and ΔE3-AdVs, cells and supernatants were harvested to perform the PFU assay. Consistent with the results of cell viability, all viruses generated plaques in HEK 293 cells; however, ΔE3-rAdVs exhibited smaller and fewer plaques (Figures 2E–F and S2). At 96h after infection, the numbers of plaques produced by Wt-AdVs were more than 10 times higher than those induced by ΔE3-AdVs.

After confirmation of attenuation in vitro, we used a mouse model to test the tissue distribution and pathogenicity of ΔE3-AdVs in vivo. In ΔE3-HAdV4 infected mice, viral gene could only be detected in lungs (Figure S3A). However, the virus could be detected in both lungs and trachea for Wt-HAdV4 infection (Figure S3B). In addition, viral loads in ΔE3-HAdV4-infected mice were lower than Wt-HAdV4 and all decreased obviously along with time. In ΔE3-HAdV7 infected animals, viruses could be detected in lungs and tracheas, while 7 days after infection, only one mouse was detectable in lung with a small amount of virus and none of them was detectable in trachea (Figure S3C). Besides, ΔE3-HAdV7 infected mice showed a lower viral load, no matter in lungs or tracheas, as contrasted with Wt-HAdV7 infection (Figure S3D). Importantly, all mice were survived without any visible signs of disease during the observation period.

### Immunogenicity study of ΔE3-rAdVs in mice

**Figure 3.**
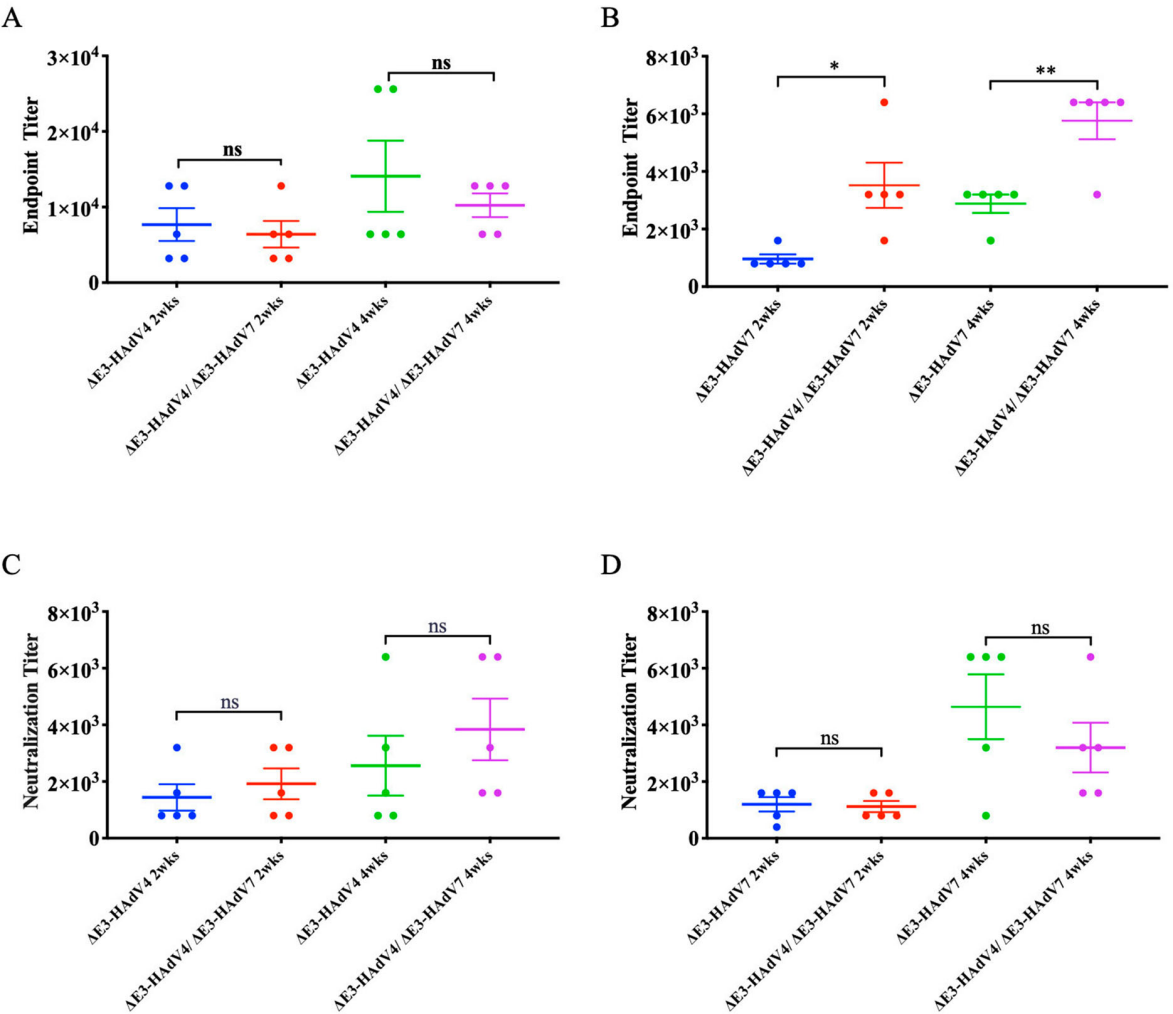
Assessment of antibody responds in BALB/c mice. Animals were immunized with ΔE3-HAdV4, ΔE3-HAdV7 or mixture of the two components for single administration, intramuscularly. Sera of mice were collected at 2 and 4 weeks after vaccination. (A) Binding antibody endpoint titres against Wt-HAdV4. (B) Binding antibody endpoint titres against Wt-HAdV7. (C) Neutralizing antibody titres against Wt-HAdV4. (C) Neutralizing antibody titres against Wt-HAdV7. Each line represents the average titre. One-way ANOVA was applied to compare the differences between groups. All data are presented as the means ± SEM. ns, no significance; *, *P* < 0.05; **, *P* < 0.01; ***, *P* < 0.001; ****, *P* < 0.0001.

**Figure 4.**
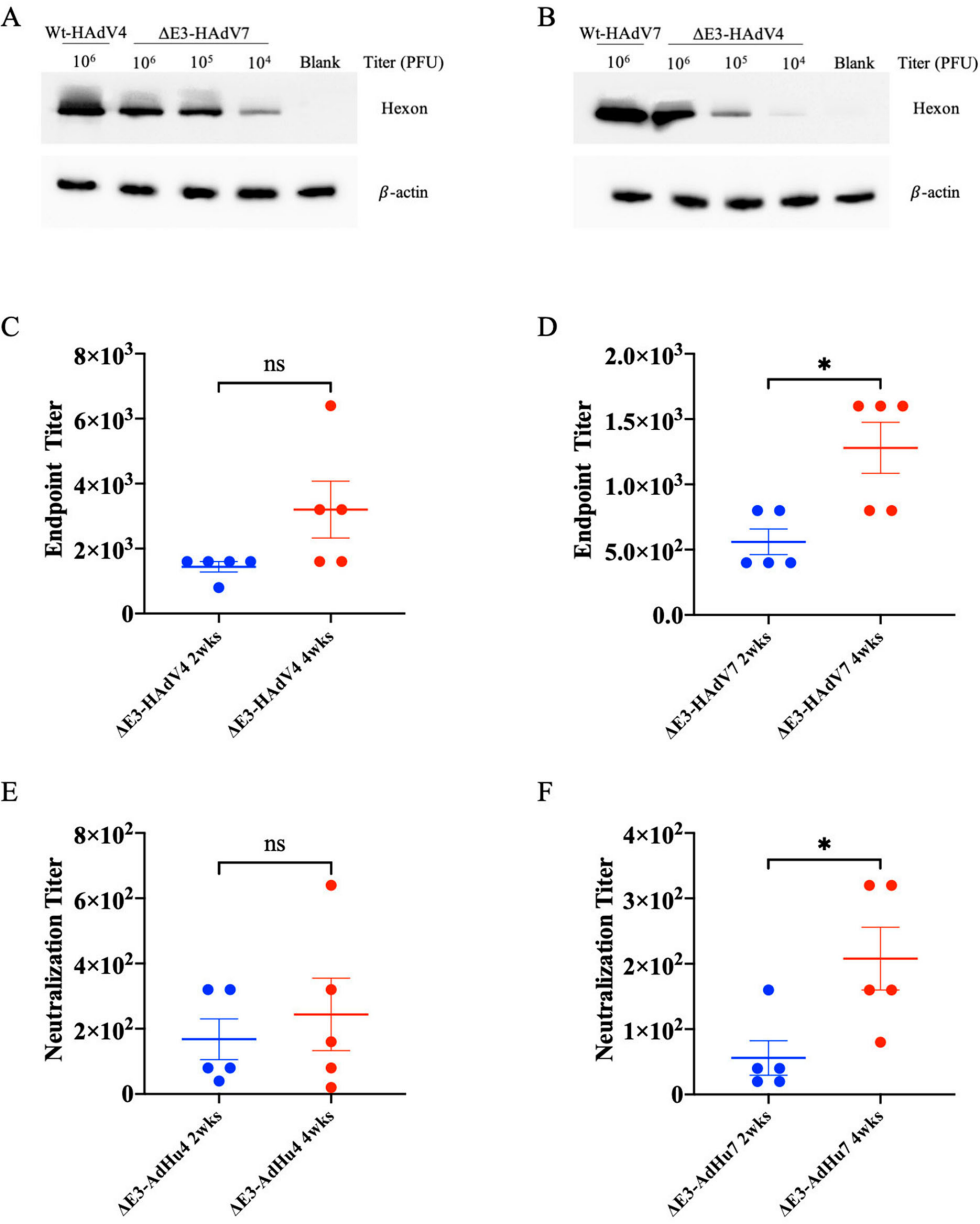
Cross antibody reaction. Sera from monovalent vaccine groups were manipulated for cross antibody reaction. (A) Wt-HAdV4 and ΔE3-HAdV7 in gradient concentration were analyzed by western blot under non-reducing condition using anti-HAdV4 sera from immunized mice. (B) Wt-HAdV7 and ΔE3-HAdV4 in gradient concentration were analyzed by western blot under non-reducing condition using anti-HAdV7 sera from immunized mice. (C) Binding antibody endpoint titres of ΔE3-HAdV4 immunized serum against Wt-HAdV7. (B) Binding antibody endpoint titres of ΔE3-HAdV7 immunized serum against Wt-HAdV4. (C) Neutralizing antibody titres of ΔE3-HAdV4 immunized serum against Wt-HAdV7. (C) Neutralizing antibody titres of ΔE3-HAdV7 immunized serum against Wt-HAdV4. Each spot represents an individual animal. One-way ANOVA was applied to compare the differences between groups. ns, no significance; *, *P* < 0.05; **, *P* < 0.01; ***, *P* < 0.001; ****, *P* < 0.0001.

To determine the immunogenicity of the novel attenuated adenovirus vaccine, BALB/c mice were immunized intramuscularly with 10^6^ PFU/mouse for ΔE3-HAdV4, ΔE3-HAdV7, or 5 × 10^5^ PFU/mouse for each component (10^6^ PFU/mouse in total). Binding affinity and neutralizing activity against wild-type adenoviruses were detectable 2 weeks after immunization in both the mono- and bivalent vaccine groups. The average BATs elicited by the bivalent vaccine were 6400 against Wt-HAdV4 and 3520 against Wt-HAdV7 at week 2, and the titres reached 10240 and 5760 at week 4 post-injection (Figure 3A,B). The NATs of the bivalent vaccine group were 1920 and 1120 against Wt-HAdV4 and 7 in week 2 (Figure 3C,D). NATs constantly increased over time and reached 3840 and 3200 by week 4 after a single immunization (Figure 3C,D). Strikingly, although the bivalent vaccine only contained half the dose of the same components as monovalent vaccine, comparable NATs as monovalent groups were still detected. Therefore, we measured the cross reaction for monovalent vaccines. Viruses can be blotted with the sera from other types of monovalent vaccine groups (Figure 4A,B), and the ELISA and neutralizing assay confirmed a cross-antibody reaction between these two types (Figure 4C–F). These cross-reactions may enhance the effectiveness of the bivalent vaccine and explain the phenomenon mentioned above. Binding and neutralizing antibody responses were stimulated after a single administration of mono- or bivalent vaccine, proving the immunogenicity of the attenuated ΔE3-AdVs in a mouse model.

### Single inoculum of bivalent adenovirus vaccine provided protection against Wt-HAdV4 and 7 challenge in mice model

**Figure 5.**
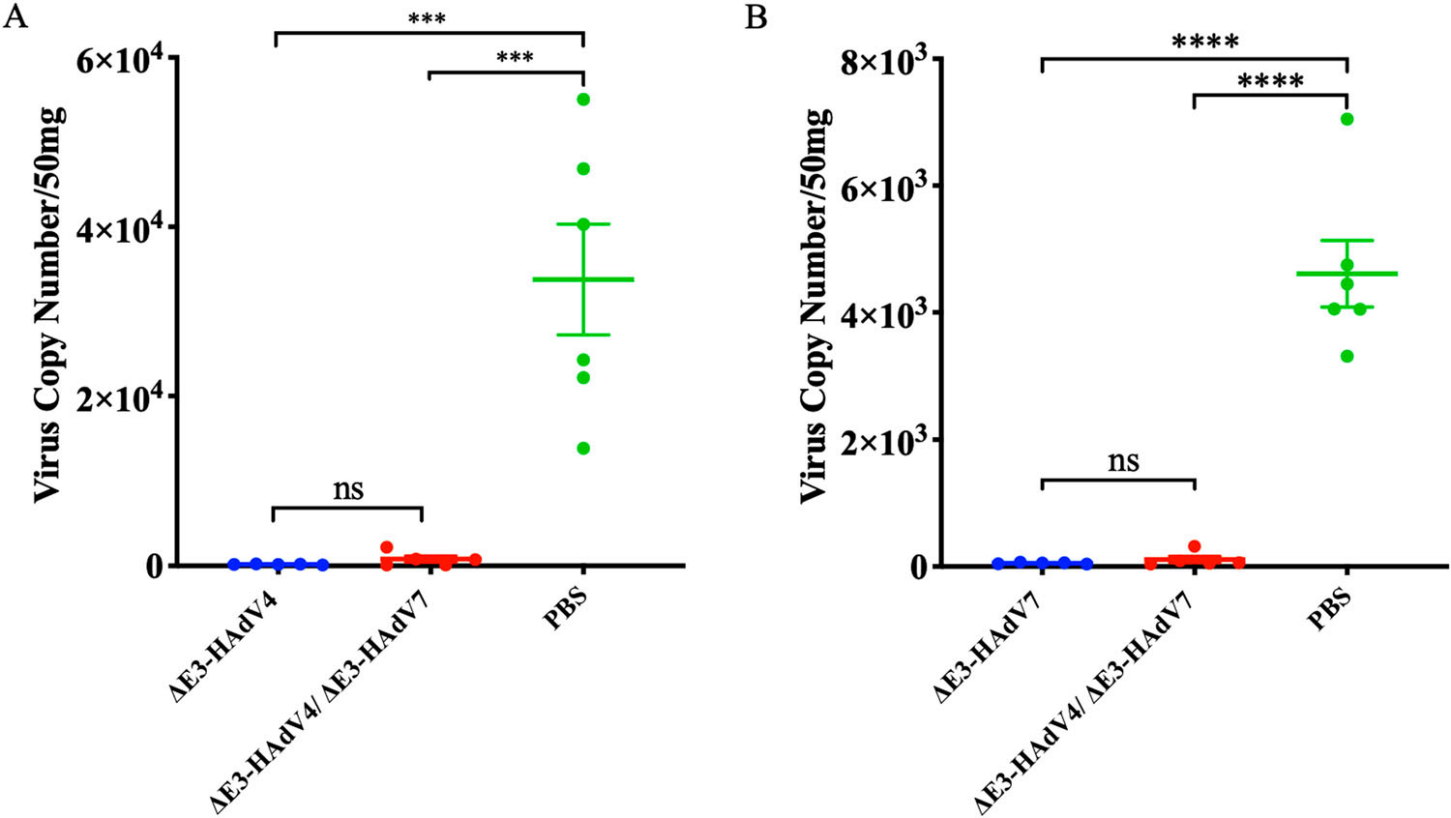
Protection against wild-type adenovirus challenge in vaccinated mice. 4 weeks after vaccination, mice were challenged. qPCR was used to determine the viral genome copies per 50 mg lung tissues on days 3 and 5 post challenge respectively. (A) Viral loads in lung tissues challenged by Wt-HAdV4; (B) Viral loads in lung tissues challenged by Wt-HAdV7. Each spot represents an individual animal. One-way ANOVA was applied to compare the differences between groups. ns, no significance; *, *P* < 0.05; **, *P* < 0.01; ***, *P* < 0.001; ****, *P* < 0.0001.

To evaluate the protective potential of ΔE3-HAdV4, ΔE3-HAdV7, or bivalent vaccine, mice were intranasally challenged with 10^6^ PFU/mouse of Wt-HAdV4 or 2 × 10^6^ PFU/mouse of Wt-HAdV7 4 weeks after inoculation. Lung tissues were collected on days 5 and 3 after challenge with Wt-HAdV4 and 7, respectively, and the viral copies in the lungs were quantified. The results showed that viral loads in the control groups were dozens of times higher than those in the vaccine groups. Approximately no viral copies were detected in the lungs of mice immunized with the monovalent vaccine for both Wt-HAdV4 and 7 after challenge. Only very limited viral copies were found in the bivalent vaccine group but were significantly lower than in the PBS control group (Figure 5A,B). However, there was no significant difference in viral loads between mice in the monovalent and bivalent vaccine groups.

**Figure 6.**
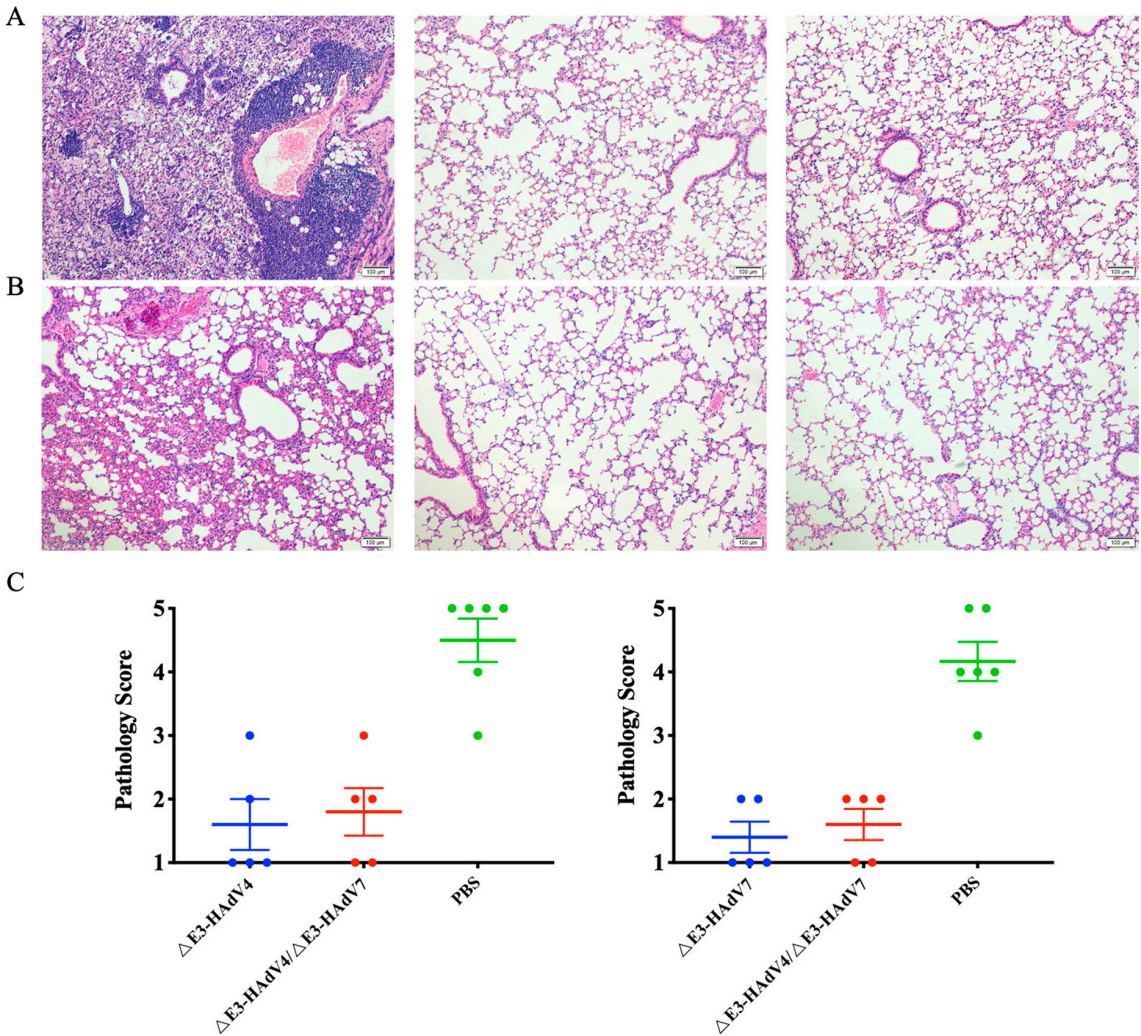
Histopathological observation and histological score of lung tissues from infected mice. Lung sections derived from mice infected with Wt-HAdV4(A) or Wt-HAdV7(B) were stained by H&E 3 and 5 days after challenge, respectively. (C) Scoring for histological changes in lungs of infected mice. Each symbol represents one mouse, and the line indicates the mean value of the group.

Histopathological analysis showed that the lung sections from mice in the vaccine groups maintained a normal structure with minimal pathological changes after challenge. In contrast, severe inflammatory exudation and alveolar fusion combined with extensive inflammatory cell infiltration were observed in the control animals (Figure 6A,B). This evidence strongly suggests that vaccination with ΔE3-AdVs was able to stimulate protective immune responses against infection by Wt-HAdV4 and 7 in mice.

### Comparation of antibody responds between ΔE3- and Wt-AdVs

**Figure 7.**
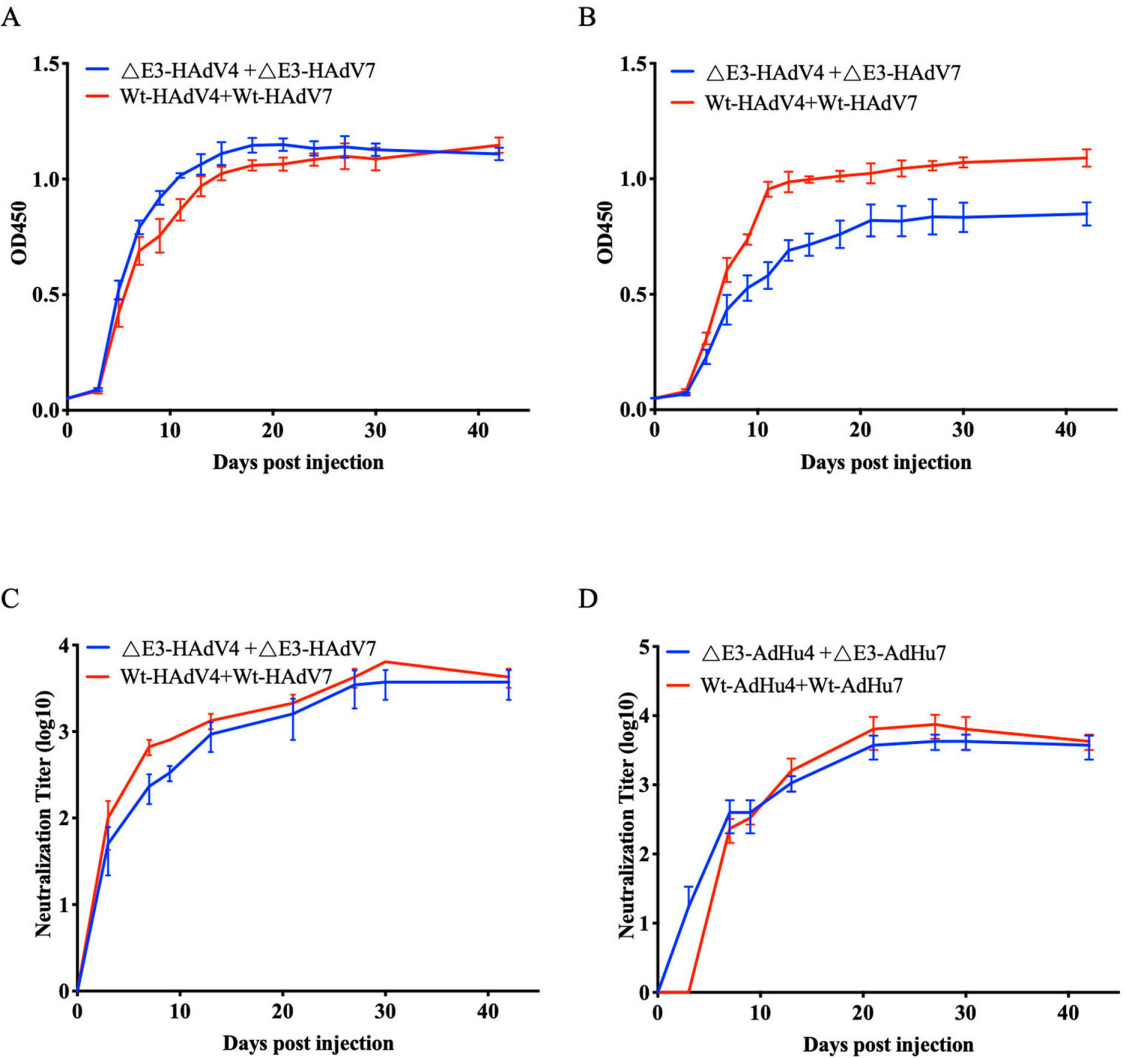
Comparation of antibody responds between E3-deleted and wild-type Ads. Mice immunized with virus of ΔE3-HAdV4 + ΔE3-HAdV7 and Wt-HAdV4 + Wt-HAdV7 were blooded constantly till 42 days. (A) Binding antibody titres against Wt-HAdV4. ΔE3-HAdV4 + ΔE3-HAdV7 vs. Wt-HAdV4 + Wt-HAdV7, ns, *P *= 0.1655. (B) Binding antibody titres against Wt-HAdV7. ΔE3-HAdV4 + ΔE3-HAdV7 vs. Wt-HAdV4 + Wt-HAdV7, **, *P *= 0.0081. Data are shown as mean absorbance among the same group (*n*=5). (C) Neutralizing antibody titres against Wt-HAdV4. ΔE3-HAdV4 + ΔE3-HAdV7 vs. Wt-HAdV4 + Wt-HAdV7, ns, *P *= 0.3620. (D) Neutralizing antibody titres against Wt-HAdV7. ΔE3-HAdV4 + ΔE3-HAdV7 vs. Wt-HAdV4 + Wt-HAdV7, ns, *P *= 0.4908. Data are shown as mean NATs among the same group (*n *= 5). Two-way ANOVA was applied to compare the differences between groups. ns, no significance; *, *P* < 0.05; **, *P* < 0.01; ***, *P* < 0.001; ****, *P* < 0.0001.

Mice were administered bivalent vaccines containing ΔE3-HAdV4 and 7 or Wt-HAdV4 and 7 via the intramuscular route. Thereafter, BATs and NATs were constantly followed until day 42 post vaccination to evaluate and compare whether binding and neutralizing activity elicited by ΔE3- and Wt-AdVs were comparable. The binding and neutralizing antibodies against Wt-HAdV4 and 7 were measured from the 3rd day after inoculation. The BATs for wild-type adenoviruses continued to increase until the 15th day post-vaccination and remained high during the ensuing period (Figure 7A,B). As for neutralizing activity, the NATs reached the highest level on the 30th day, and then decreased in the Wt-AdVs vaccine group (Figure 7C,D).

Regarding to the immune responses to HAdV4, there was no significant difference in the binding and neutralizing antibody levels between Wt-AdVs and ΔE3-AdVs bivalent vaccines (Figure 7A,C). The NATs of the ΔE3-AdVs group showed no decreasing tendency at the end of the detection (mean titre of 3733) (Figure 7C). However, the binding affinity against Wt-HAdV7 in mice in the ΔE3-AdVs group was lower than that in the Wt-AdVs group (Figure 7B), although the neutralizing activity was equivalent between the two groups (mean titres of 3733 and 4267, respectively) (Figure 7D). Similarly, the NATs reached a peak at 27 days and then began to decrease in the Wt-AdVs group, whereas NATs in the ΔE3-AdVs group remained stable at the end of detection.

## Discussion

Subspecies B adenovirus HAdV-7 and subspecies E adenovirus HAdV-4 have occurred epidemically and induced acute respiratory diseases (ARD) [40]. In particular, among populations in relatively closed environments, both for infants and adults, viruses can spread faster and cause worse symptoms, even death [5,6,41,42]. In addition, the increase in population density, more frequent migration, accelerated aging trend, and increased number of newborns, all of which contribute to the circulation and severity of adenoviruses. Several studies have demonstrated that mutations, gene recombination, patients’ preexisting conditions, and multiple adenovirus co-circulation all increase the severity of adenoviral infection [14,40,43–46]. Although vaccination has been proven to be effective in preventing adenoviral infection, only one orally administered live adenovirus vaccine is available; however, it is limited to the U.S. military [21,26]. Zhou et al. constructed several adenovirus vaccine candidates targeting HAdV3, 7, 14, and 55, respectively, using a replication-deficient adenoviral vector derived from HAdV3 [47–49]. This study provides an alternative option for different pathogens, vaccine types, vaccine designs, and construction strategies.

This study constructed two E3 regions deleted recombinant adenoviruses (rAdVs) derived from Wt-HAdV4 and 7, termed ΔE3-HAdV4 and 7. The two ΔE3-AdVs were successfully rescued in HEK293 cells. Western blot results confirmed that hexons, the major targets of neutralizing antibodies, are present on the surface of viral particles (Figure 1). After E3 gene deletion, ΔE3-AdVs showed a lower peak titre and reduced replication efficacy (Figure 2). Additionally, cells infected with Wt-AdVs showed an extensive decrease in viability compared with cells infected with ΔE3-AdVs simultaneously (Figure 2). Mouse infection model proved that the recombinant virus with E3-deletion decreased in viral loads in tissues compare to the wild-type virus (Figure S3), and the virus can be cleared over time. Based on these data, we concluded that Wt-HAdV4 and 7 can be attenuated by E3 genes deletion and have potential application value as live attenuated vaccines.

E3 genes are considered dispensable for viral replication and cytopathogenicity. This study found that deletion of the E3 gene contributed to the attenuation of HAdV4 and 7. A possible explanation for this might be that E3 gene products are required for optical replication of HAdV4 and 7, although this is not necessary for HAdV5 based on previous studies. In addition, several studies have shown that E3 gene products can regulate late gene expression, and deletion of the E3 genes affects replication efficiency. In addition, the presence or overexpression of the E3 gene can enhance the cytotoxicity of adenoviruses in *vivo* or in *vitro* [50–53]. These results suggest that E3 genes may have multiple functions and are related to viral replication and virulence.

We further evaluated the efficiency of LAV in the mouse model (Figures 3–6). This shows that a single intramuscular dose of LAV consists of ΔE3-HAdV4, ΔE3-HAdV7 can induce sufficient immunity responses with high BATs and NATs (Figure 3). In addition, a cross-antibody reaction was observed between the two subspecies of HAdVs (Figure 4). Viral loads of lung tissue and histological observations also proved that LAV protects mice against adenovirus infection (Figures 5 and 6). In contrast to Wt-AdVs, LAV showed comparable neutralizing antibody titres and durability (Figure 7). Although the NATs in the Wt-AdVs group were slightly higher until approximately 30 days post-vaccination, they rapidly dropped to the same level as the LAV group at the end of detection (42 days) (Figure 7). Furthermore, the NATs of the LAV group reached a plateau approximately 30 days after inoculation and remained stable at the same level until the end of detection. The reduction of viral replication and attenuation may be why NATs in the LAV group were slightly lower than those in the WT group. The wild-type adenovirus with full replication ability may induce an excessive immune response which is also a threat to the safety of vaccines, and then be eliminated by the immune system more quickly, resulting in a decrease in antibody titres.

One of the concerns regarding the development of LAVs is their stability since live-attenuated viruses may have the risk of reversion to virulence during passage [54]. To assess the stability of ΔE3-HAdV4 and ΔE3-HAdV7, we passaged two viruses in HEK293 cells for 12 generations. After passage, we found that two ΔE3-HAdVs remained stable without detectable genetic instability by enzyme digestion or alterations in growth curves and plaque-forming ability (data not shown). These results indicate that the risk of reversion to virulence of ΔE3-AdVs is low since we completely modified the genomes of the virus, which guarantees the safety of its usage as a potential LAV.

However, no suitable permissive animal model can be achieved for Wt-HAdV4 and 7 replication and infection. Therefore, mice infected with HAdVs showed no evident symptoms, including high body temperature or weight loss. The viral load in the lungs of mice is also low and decreases with prolonged infection. Therefore, it is urgent to develop a permissive animal model for HAdV infection and vaccine evaluation in future studies.

In summary, our results suggest that E3-deleted human adenovirus types 4 and 7 have potential as an adenovirus vaccine that can induce a robust humoral immune response and provide protection for vaccine-immunized mice against viral infection. This study also presents a new strategy for developing LAVs for different adenovirus serotypes.

## Supplementary Material

Figure_S3.tif

Figure_S2.tif

Figure_S1.tif
