# Supplementary figures and images for: A bivalent live-attenuated vaccine candidate elicits protective immunity against human adenovirus types 4 and 7
Source: Emerg Microbes Infect. 2021 Sep 27;10(1):1947–59. doi: 10.1080/22221751.2021.1981157 (PMC8477930; doi:10.1080/22221751.2021.1981157)

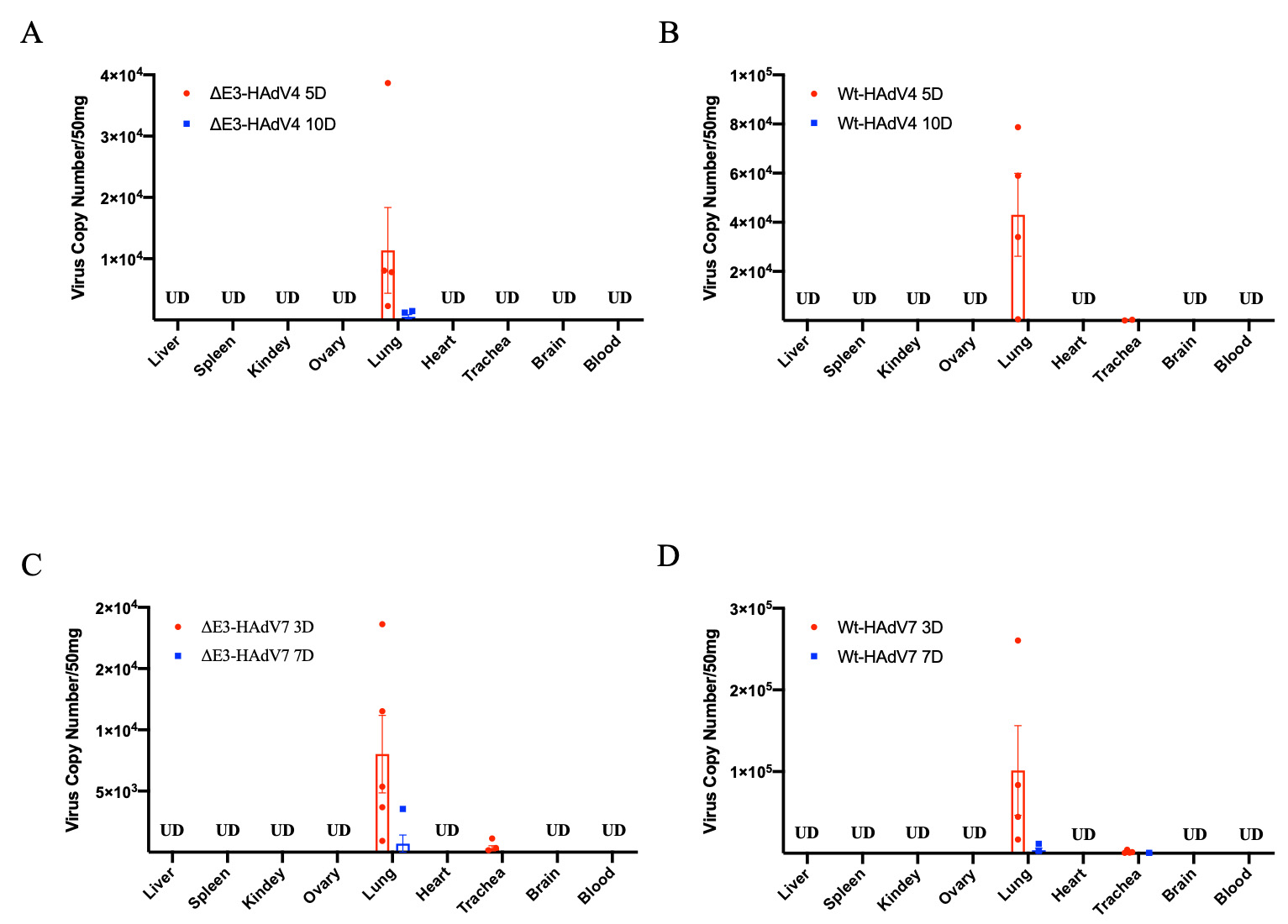

Supplement: Figure_S3.tif [file TEMI_A_1981157_SM7399.tif]

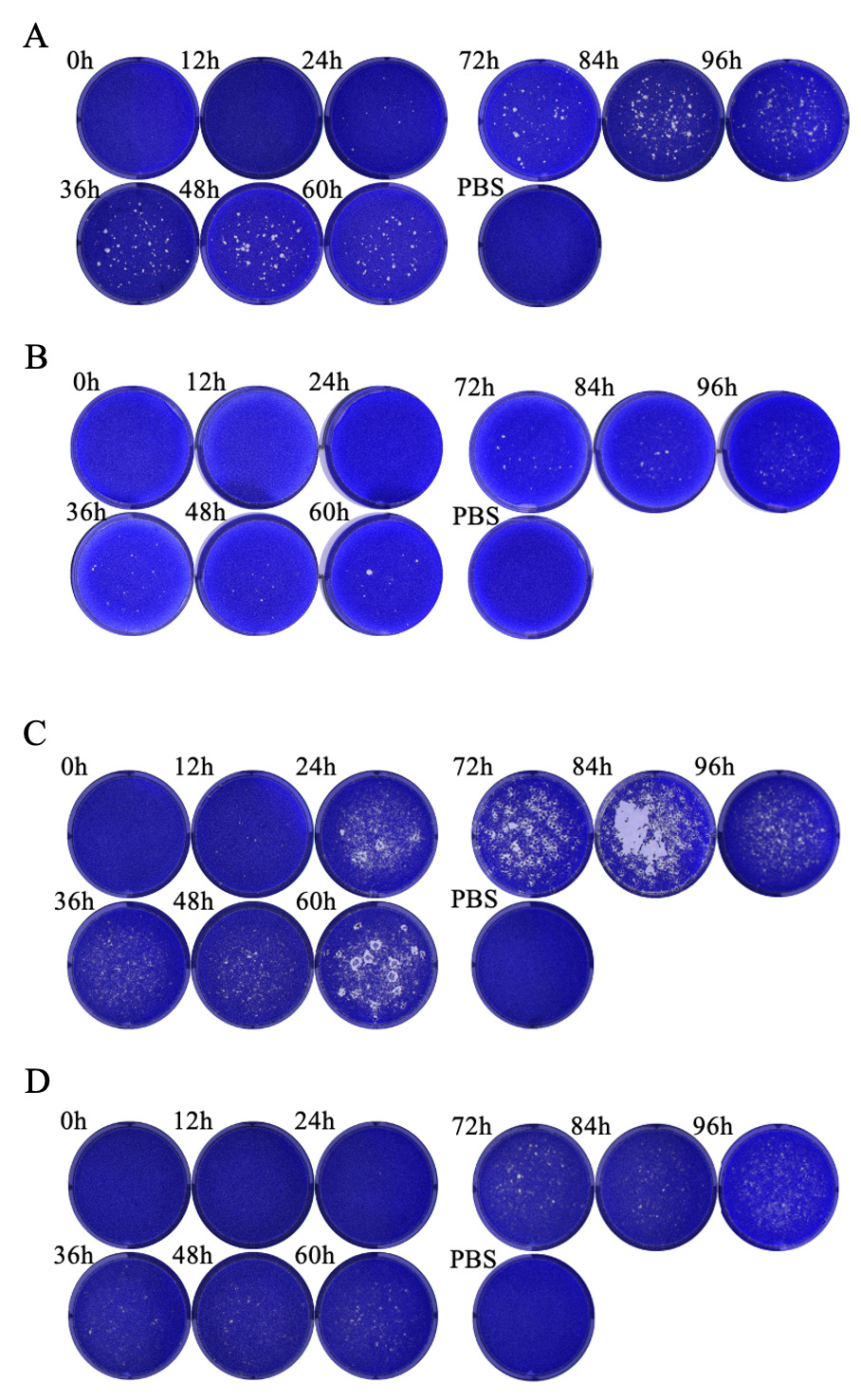

Supplement: Figure_S2.tif [file TEMI_A_1981157_SM7398.tif]

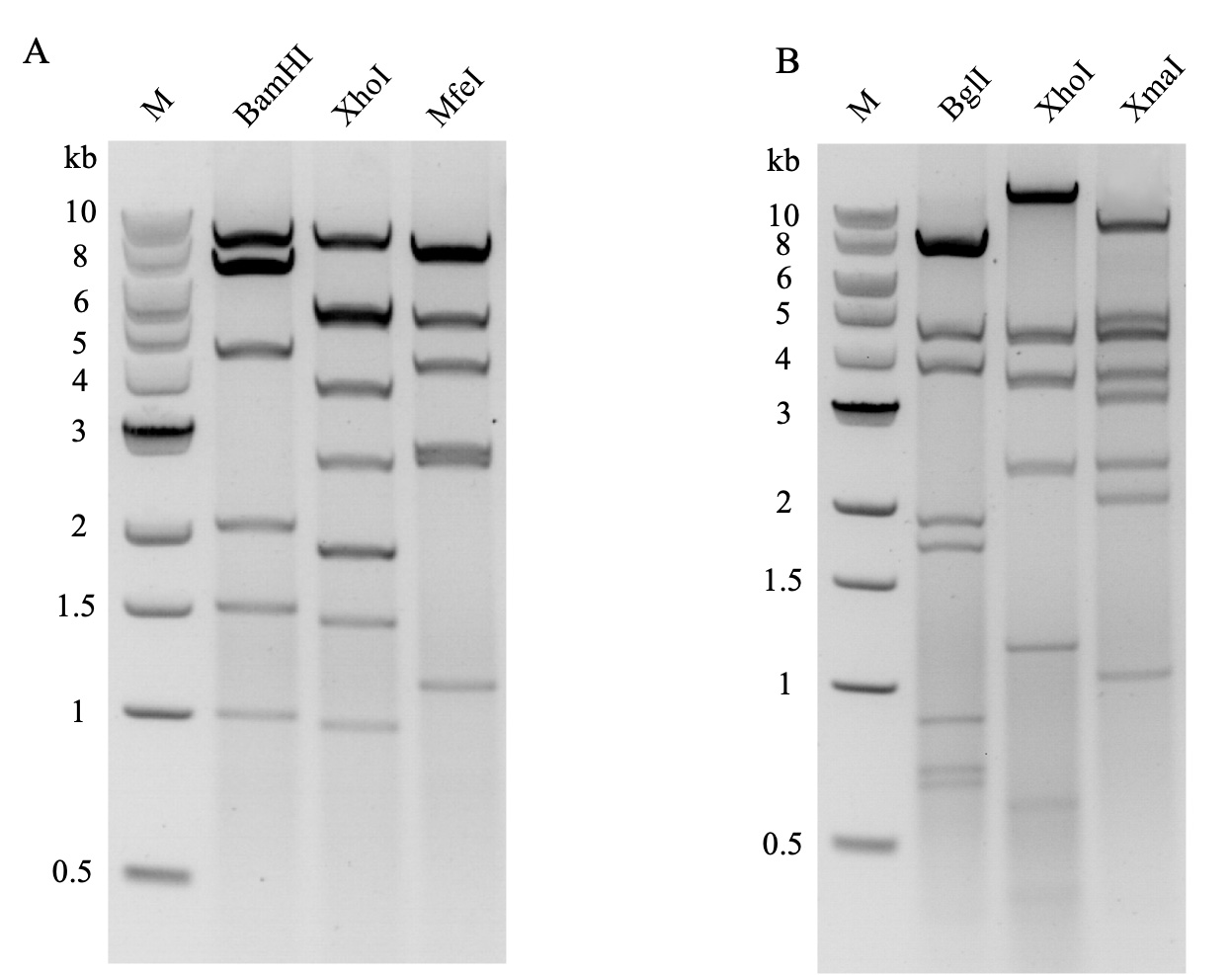

Supplement: Figure_S1.tif [file TEMI_A_1981157_SM7397.tif]
